# A decision support framework for prediction of avian influenza

**DOI:** 10.1038/s41598-020-75889-7

**Published:** 2020-11-04

**Authors:** Samira Yousefinaghani, Rozita A. Dara, Zvonimir Poljak, Shayan Sharif

**Affiliations:** 1 School of Computer Science, University of Guelph, Guelph, ON Canada; 2 Department of Population Medicine, Ontario Veterinary College, University of Guelph, Guelph, ON Canada; 3 Department of Pathobiology, University of Guelph, Guelph, ON Canada

**Keywords:** Data mining, Data processing, Machine learning, Predictive medicine

## Abstract

For years, avian influenza has influenced economies and human health around the world. The emergence and spread of avian influenza virus have been uncertain and sudden. The virus is likely to spread through several pathways such as poultry transportation and wild bird migration. The complicated and global spread of avian influenza calls for surveillance tools for timely and reliable prediction of disease events. These tools can increase situational awareness and lead to faster reaction to events. Here, we aimed to design and evaluate a decision support framework that aids decision makers by answering their questions regarding the future risk of events at various geographical scales. Risk patterns were driven from pre-built components and combined in a knowledge base. Subsequently, questions were answered by direct queries on the knowledge base or through a built-in algorithm. The evaluation of the system in detecting events resulted in average sensitivity and specificity of 69.70% and 85.50%, respectively. The presented framework here can support health care authorities by providing them with an opportunity for early control of emergency situations.

## Introduction

Avian influenza virus, with a natural reservoir in aquatic wild birds^1^, causes a disease with high economic impacts on the poultry industry throughout the world. Moreover, the epidemics of avian influenza remain a major threat to animal and human health, highlighting the need for the development of tools that assist in decision making. Surveillance and prediction of avian influenza emergence can help in responding to infectious disease emergencies by providing advance knowledge of the location, timing and intensity of disease events. Events are defined as unusual events that might signal an outbreak^2^. Advance knowledge provided by surveillance systems assists policy makers in selecting appropriate measures to contain virus spread. Given the complexity of disease introduction and transmission mechanisms, studies have usually considered different aspects of information. However, there is a small group of epidemiological studies that use decision making methodologies involving multiple factors, options and data sources^3^.

There have been efforts to explore the critical risk factors for avian influenza infection, such as environmental dynamics, wild bird migratory routes, bird trade routes, and water or rice lands^4–6^. The most important risk factors have been recognized and used to build forecasting models including spatiotemporal visualisation, time-series, and data mining models. These models could facilitate the process of decision making by predicting the time and location of epidemics. In addition, social media, search engines, and news contents have been used as an alternative to official records of infectious diseases^7–9^. Digital sources of data have been used to monitor the spatial spread of disease as time progresses. The insights from digital surveillance systems may support decision makers by providing them with early warnings of epidemics.

### Decision support system (DSS)

Decision support systems can assist policy makers to take effective management decisions for containment of infectious disease outbreaks. Broadly speaking, a DSS is a digital system that assists in the process of making decisions^10^. A traditional DSS is defined as a system that supports managerial decisions in semi-structured situations, which aims to enhance the ability of decision makers, rather than replace their opinion^10^. A traditional DSS is, generally, composed of: databases that are managed by a database management system, a model management that provides quantitative models with analytical capabilities, and a graphical user interface (GUI) or a dashboard^11^.

Additionally, over time, DSSs have evolved to more sophisticated systems known as knowledge-based DSS. Knowledge-based systems make use of a variety of models, including artificial intelligence^12^. In fact, a knowledge-based system is not only a repository for rules, but it is also a tool to deliver intelligent decisions by utilizing data mining and artificial intelligence methods^10^. A typical knowledge-based DSS includes five main components, namely: data, model, knowledge base, interface engine and user interface.

Typically, systems that have been designed to assist decision makers in the field of infectious disease range from online information systems^13^ to model-driven systems^14,15^ and machine learning based systems^16,17^. The information system introduced by Li and colleagues^13^ exploited mobile and wireless infrastructures to facilitate the collection, exchange, and visualization of information. The extracted information then could be queried and analyzed by experts in the field to make proper decisions. The next group of systems, have used statistical simulation models to provide required information for experts^14,15^. In this type of systems, experts can classify or cluster the information or they can search the parameter space to find an optimum control scenario. In the last group of DSSs^16–18^, machine learning algorithms and online analytical processing of data (OLAP) have been utilized in order to extract useful knowledge. The outcome knowledge can then assist in identifying the future geotemporal occurrence of disease and potentially containment policy making.

Knowledge fusion is an effective method to enhance the efficiency of a decision support system^19^. In several studies, data from various domains have been combined to gain more detail and reliable information on disease patterns^16,18,20^. In a study by Chae et al.^18^, it was shown that deep learning methods perform much better than time-series approaches for predicting infectious diseases 1 week into the future. The analysis was applied to big data including social media, environmental factors, search queries and disease occurrence data. The study by Chae and colleagues concluded that deep learning models can reduce the reporting delays in the existing surveillance systems and cut down costs. In another research, Sun et al.^20^ integrated information from outbreak data, genetic sequences and several risk factors in order to gain more accurate information on identifying high-risk areas. Global spatial patterns of highly pathogenic avian influenza were extracted using logistic spatial autoregressive, local k function, phylogenetic tree analysis and Dampster–Shafer evidence^21,22^ models and concluded that Dampster–Shafer theory was more reliable and robust than other models.

Similarly, case data, population statistics and weather conditions were utilized in statistical models to forecast dengue weekly incidence^16^. The least absolute shrinkage and selection operator (LASSO) algorithm was proven to gain a better performance compared to linear regression and time-series. The LASSO model was used for its high forecast accuracy, but was not able to interpret outbreak predictions. For example, the explanation of why the model forecast a large epidemic in a particular year to stakeholders was not possible.

Fusion of information in decision support systems was performed in a different way in a study conducted by Texier and colleagues^23^. In this study, the decisions of multiple outbreak detection algorithms were combined using several methods including Majority Voting, Logistic Regression, Decision Tree and Bayesian Network. Finally, Bayesian Network was suggested to be used for decision fusion as it gains at least a performance equal to the best of the individual algorithms. In another study^24^, a framework was designed to integrate knowledge from different domains for avian influenza outbreak identification. Dempster–Shafer theory was employed to integrate findings of three sets of different methodologies including phylogenetics, spatial statistics and epidemiological analysis. Authors found that integrating all three analyses resulted in a higher level of corroboration than when only one methodology was used.

Despite the efforts that have been made to develop decision support systems for controlling infectious diseases, there are still some challenges that remain unaddressed. There is a limited uptake of the decision support systems by policy makers, which probably means the existing decision support systems have not been designed according to the needs of policy makers. Moreover, the presence of multiple forecast systems with different goals makes it difficult for decision makers to choose one. Another challenge is the lack of transparency of decisions in the existing systems, which is a crucial requirement in policy making. The current study aimed to examine solutions to these challenges.

### Motivation

Current infectious disease response programs have several limitations that need to be addressed. Time plays a key role in infectious disease management and control. To facilitate management in disease emergencies, making rapid policy decisions is crucial. Early warning systems are defined as timely surveillance systems that collect information on diseases with epidemic possibilities in order to plan intervention policies^25^. The short amount of time in disease emergency situations neither allows for developing, parametrizing and interpreting new models nor in-depth data reviews by epidemiologists^25^.

In addition, decision makers continuously rely on epidemiological models with many assumptions to understand the future progression of disease outbreaks^27^. The outcome evidence of these models can be interpreted in multiple ways that may lead to inaccurate understanding of situations.

Another problem that could impede response programs is modelling fragmented and incomplete datasets^28^. A comprehensive data management is necessary for successful data integration and knowledge extraction^29^. Studies that employ only a single domain knowledge can lead to one-sided knowledge and miss important information^24^. Moreover, risk assessment studies in epidemiology are usually restricted to health events that have already occurred^30^. However, retrospective studies alone cannot be used for real-time evaluations.

In the present study, we proposed and evaluated a comprehensive and multi-scale framework for avian influenza event prediction. The main goal of the framework was to integrate patterns from digital and spatiotemporal surveillance systems and answer three types of questions: (I) Descriptive questions regarding avian influenza event in the past; (II) Predictive questions regarding the future risk of avian influenza at country-level; and (III) Predictive questions regarding the future risk of avian influenza at provincial-level. We incorporated knowledge concerning disease occurrence history, environmental conditions, migratory bird distribution, poultry density and social media into the framework. Given the three questions that policy makers might ask, a procedure was developed to respond to those questions.

As discussed earlier, decision support frameworks should suit the needs of practitioners and policy makers^31^. In this current proof-of-concept study, a link between decision makers and modellers was build by answering some risk-predicting questions. These questions could be asked by decision makers in order to prepare for and respond to epidemics. The second innovation lies in providing transparent predictions, which means animal health officials can find the reasons behind the predictions made by the system. This is because the rule-based models used in the system are human-interpretable and could greatly facilitate interpretation of decisions. An additional novelty of the system is the continuous and comprehensive data collection, integration and analysis of avian influenza information, which allows rapid and precise results^32^.

## The proposed framework

### System description

**Figure 1:**
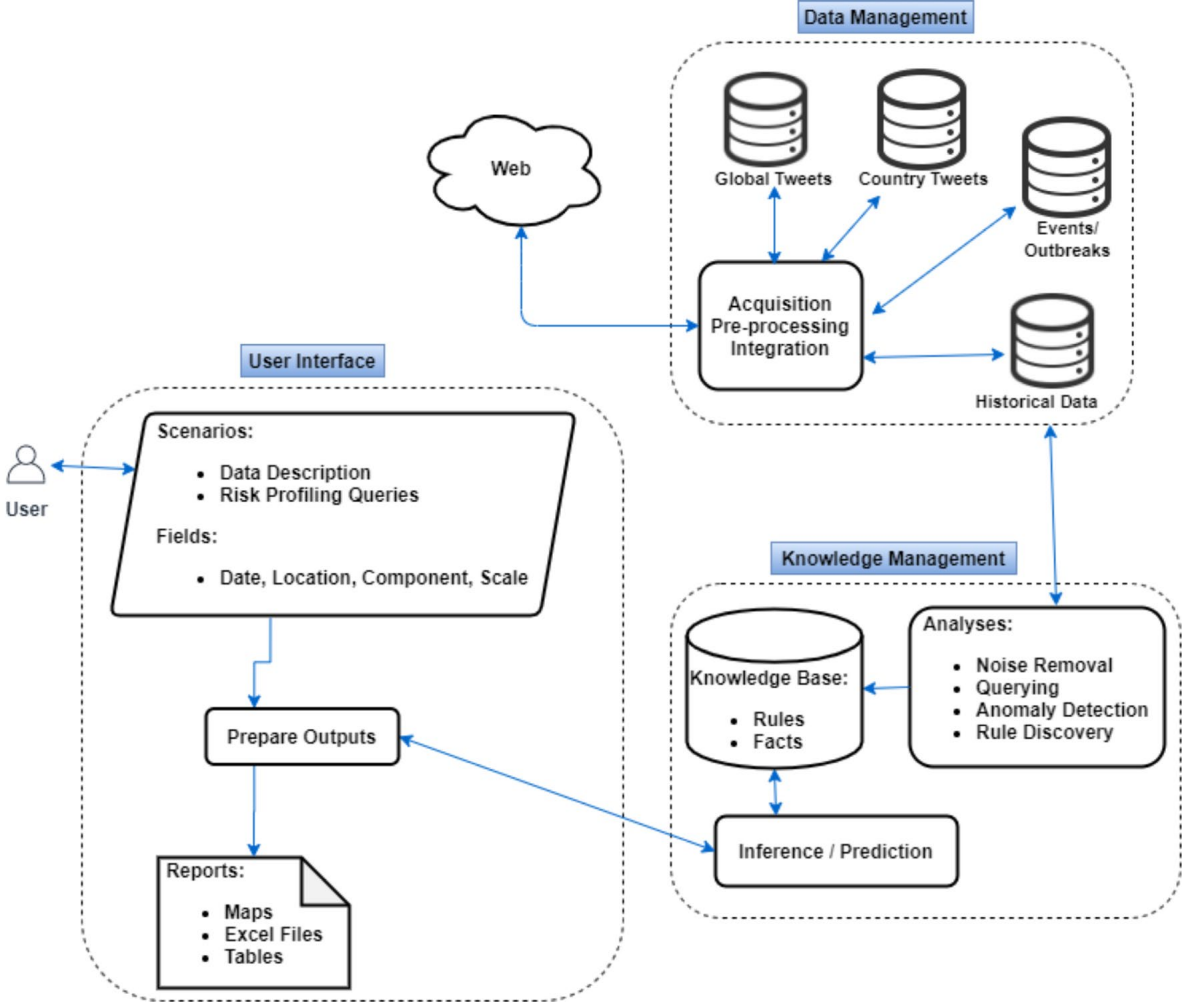
Framework architecture.

The framework presented here is an extension of previous works and is intended to assist in the early detection of avian influenza events. The framework consists of three parts: data management, knowledge management and user interface. First, several data sources were collected, pre-processed and stored. Then, rules and facts were extracted from the data and stored in a knowledge base. Rules are used as a technique for knowledge representation in a system^33^ while facts are known information about data. Finally, the knowledge base was used to respond to questions regarding the degree of avian influenza risk at different geographical scales. In the present study, the risk is defined as the likelihood of disease occurring for the first time or continuous events in a region. An overview of the framework is given in Fig. 1. Figure 1 depicts how different elements in each part are related to one another.

### Data management

The data management part was responsible for several operations including data acquisition, pre-processing and integration. Data was collected from Twitter and spatiotemporal data sources. Subsequently, pre-processed techniques such as data cleaning and transformation were applied to raw data to transform it to a useful format for the knowledge management.

#### Spatiotemporal covariates data

We identified several explanatory variables of avian influenza including temperature, precipitation, humidity, wind speed, pressure^34^, chicken density, duck density^35^ and waterfowl density^36^. These risk factors have been proven to correlate with avian influenza events in previous studies^37,38^. The corresponding data sources including climatic conditions, geographic extent of migratory bird species, distribution of poultry, and disease historical records were integrated in a spatiotemporal dataset. Integration was performed considering a spatial and temporal resolution of 1-degree × 1-degree and 1-week, respectively. Then, all explanatory variables and response variable were adjusted in the defined resolution.

Predictor variables with different spatial and temporal resolutions were adjusted with respect to the defined spatial and temporal resolution. Variables with temporal scale less than a week and spatial scale less than a cell were averaged. Conversely, when the resolution was lower than a cell or a week, we repeated the same values for all the cells or weeks that fit into that resolution.

Darksky API was used to return the observed daily weather conditions given a specified date in the past and a location point. The Gridded Livestock of the World offered GeoTIFF format files that were converted to longitude-latitude-value format and then imported to a designed database. Birdlife species data included shapefiles that could be visualized by geographical information system (GIS) software such as ArcGIS. We filtered polygons related to 133 duck species, and in the field called ‘bird_existence’ in the database, we specified whether each cell was inside a bird polygon or not. Other information on these datasets is presented in Table 1.

**Table 1:** Sources of data.

Data source	Description
Dark Sky API	The API offers several climatic variables including temperature, humidity and wind speed. We automatically collected the variables that have been frequently used as risk factors of avian influenza. The ‘Time Machine Requests’ API offered by Dark Sky^34^ was used to retrieve weather information given latitude, longitude and time parameters
BirdLife-species	The data provides geographic extents of species distribution ranges and is available in the Environmental Systems Research Institute (ESRI) Geodatabase formats^36^
Gridded Livestock of the World (GLW3)	Food and Agriculture Organization (FAO) has developed the GLW3, in which the global distribution of chickens and ducks in 2010 is expressed by the total number of birds per pixel (5 min of arc)^35^
EMPRES-i	FAO’s Emergency Prevention System (EMPRES) offers a web-based application in order to facilitate the organization and access to disease data at various geographical scales which supports veterinary services^39^

#### Global Twitter data

Twitter data as a source of disease surveillance can bypass formal information channels and enhance the speed of control actions. To collect global (i.e. country-level) tweets, a crawler was used to visit Twitter on a per minute basis. Several keywords regarding avian influenza were fed into the Twitter Search API and posts were continuously stored in a database. The dataset, in total, contained 209,000 observations, which were collected over 18 months. Subsequently, tweets were filtered and geo-located using their context and re-tweets observations were removed. Subsequently, tweets were geo-located based on their content and irrelevant ones were filtered using a semi-supervised classification. Additionally, duplicate tweets (e.g., re-tweets) were removed from data. The details of data collection and pre-processing of data was previously described^40^.

**Figure 2:**
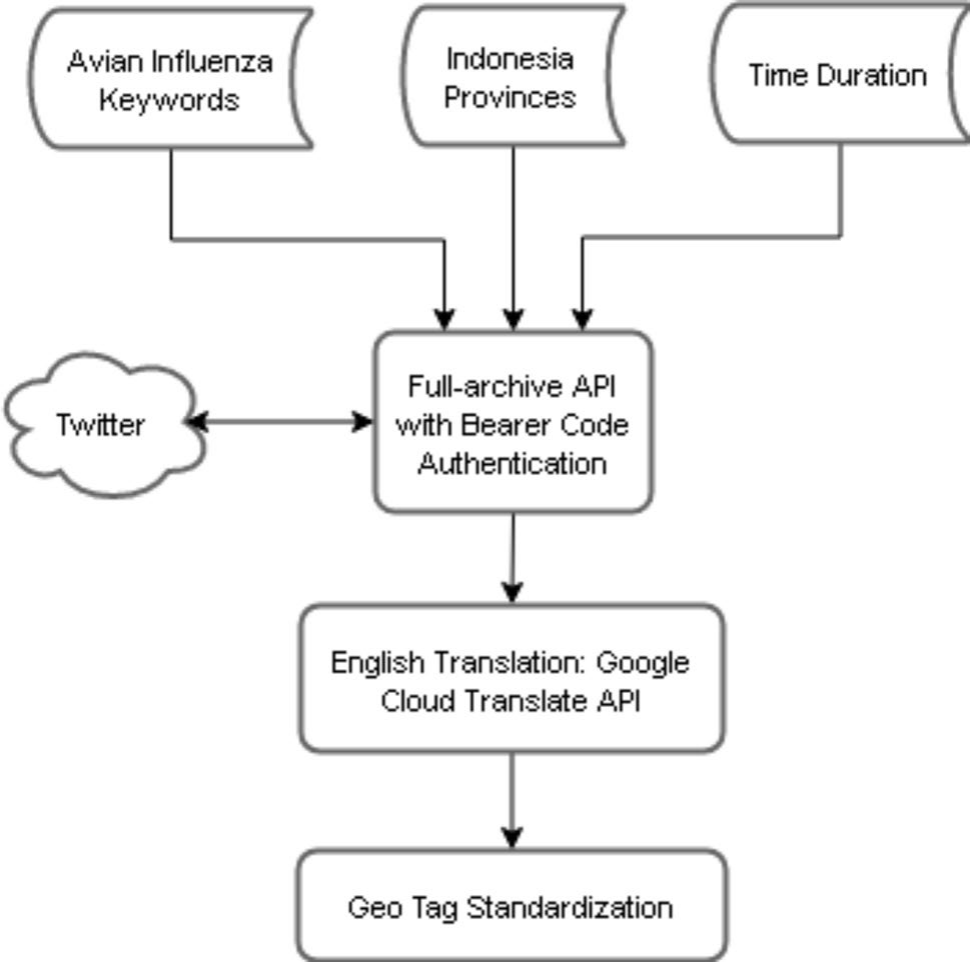
Country-level data collection pipeline.

#### Country-scale Twitter data (Indonesia)

A general overview of the provincial-level Twitter data collection pipeline is provided in Fig. 2. Employing the full-archive endpoint of Twitter premium search API, tweets regarding avian influenza for various provinces in the country of Indonesia were requested using a combination of keywords and operators. For example, for Java province in Indonesia, we used the following input: “flu burung Java OR birdflu Java OR H5N1 Java OR H7N9 Java OR bird flu Java OR H5N2 Java”. Tweets were collected for the year 2016, which resulted in a total of approximately 5,000 observations. We selected this period since it was matching to the duration of spatiotemporal covariates data and also due to restrictions on the number of requests in Twitter API.

After fetching data from Twitter and storing it in a dataset, tweets were translated into English from the language spoken in that specific country (i.e. Indonesian Language) utilizing Google Cloud Translate API. Given the translated content, we tagged each tweet with a standard list of province names.

#### Ground truth data

To collect country-level disease events, we utilized a programmed robot to visit OIE web pages^41^ every four hours. The robot filtered and stored AI-relevant reports including 58 immediate and 382 follow-up notifications for the same duration that global-scale tweets have been collected. Besides, the information on avian influenza events for provincial-level (for Indonesia) was obtained from the Emergency Prevention System for Animal Health (EMPRES-i)^39,42^.

### Knowledge management

**Figure 3:**
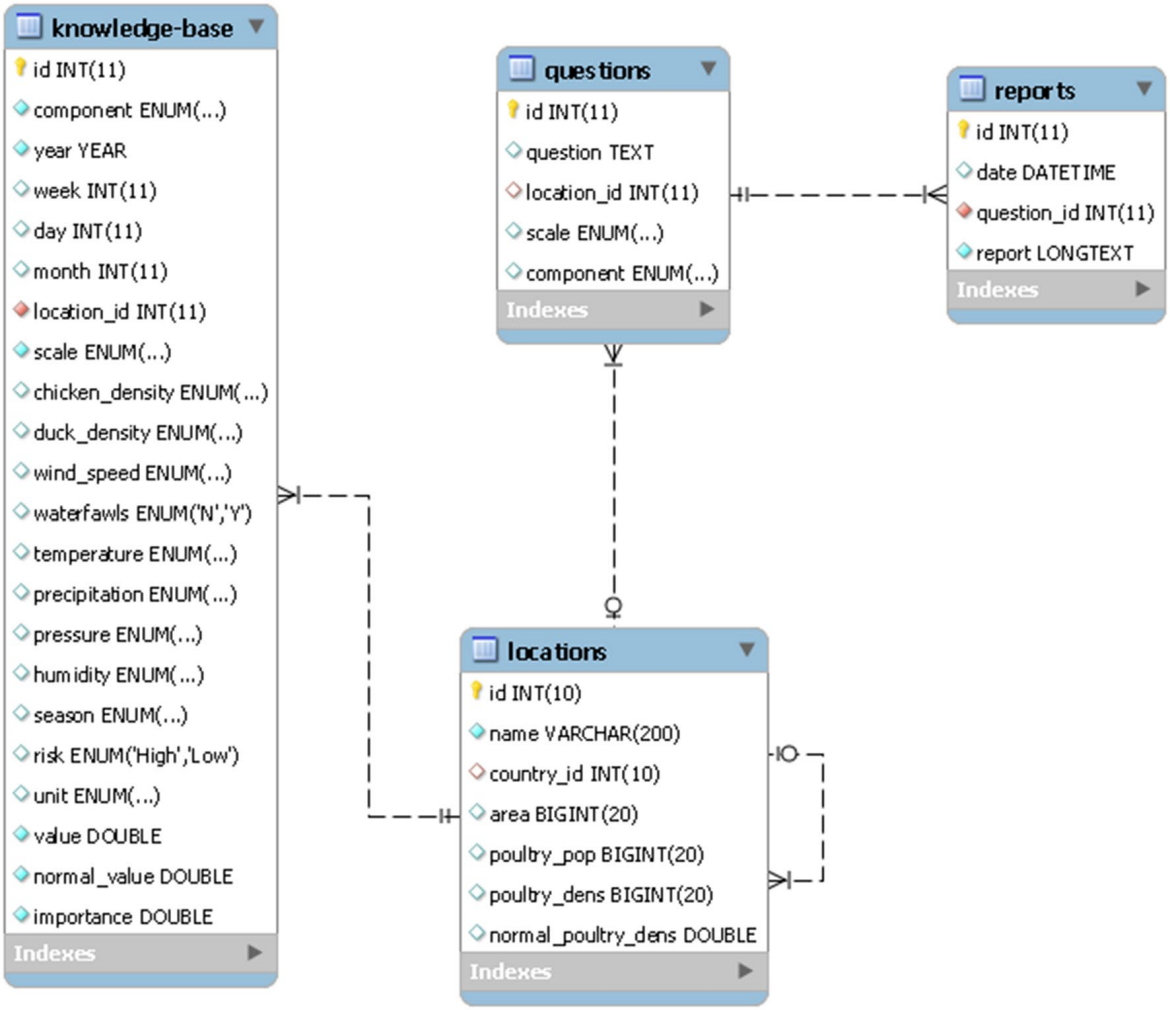
DSS database schema.

The knowledge management part of the system aimed at turning data into insights that facilitate decision making. As illustrated in Fig. 1, the knowledge management component of the system consists of a knowledge base, analysis methods and risk prediction. The knowledge base was designed as a table called ‘knowledge-base’ (see Fig. 3) to store rules and facts derived by applying a set of analyses to the collected data. The knowledge base was then used in answering the questions that end users might ask. SQL queries, noise removal, anomaly detection and rule discovery were analysis methods used in the knowledge management.

The historical events of avian influenza were stored as facts with time, location and magnitude elements. Also, several analyses have been performed on Twitter data in order to extract patterns and store them in the knowledge base. For instance, the irrelevant content was filtered out using a semi-supervised classifier. Subsequently, Seasonal-Hybrid Extreme Studentized Deviate (SH-ESD) algorithm was employed to identify spikes from daily time-series of tweets. Then, the patterns containing time, location and magnitude elements were stored in the knowledge base. Moreover, analyses on the spatiotemporal data were performed to derive patterns in the form of ‘if-then’ rules from RuleFit and FP-Growth models. These rules were built using explanatory variables, rank, time and location elements.

Descriptive and country-level predictive questions were addressed using the patterns stored in the knowledge base. However, for the predictive questions at the provincial-level, it was necessary to perform additional analysis. The ‘knowledge-base’ table and the spatiotemporal dataset^43^ were used to calculate the weekly risk of disease events for each province. Algorithm 1 presents the pseudo-code for a step-by-step process of the risk calculation.
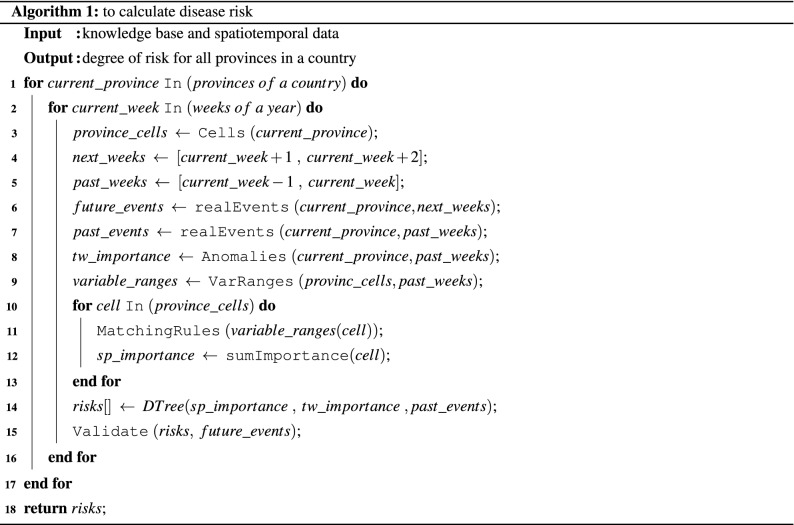


To calculate the future risk of disease occurrence for a province, a multi-class decision tree classification was applied to risk degrees obtained from the Twitter component, spatiotemporal component and past disease events. Multi-class tasks assume that each example is assigned to one and only one label. Decision tree classifiers are frequently used in classification problems with a good accuracy compared to other machine learning algorithms^44^. A decision tree is composed of rules extracted from a training set. Feature values at each branching point of the tree are used to split the data. Subsequently, the tree is traversed from the root to leaves with each branching point determining the direction that needs to be followed. Finally, the new instance is assigned with the associated label of the tree leaf that is reached^45^. Decision tree classifier was implemented here with the help of sklearn python library and a grid search parameter setting.

The risk of Twitter and spatiotemporal components was categorized into very low, low, medium, high and very high while the risk of past and future disease events was represented by low, medium and high levels. Table 2 shows the thresholds used to discretize the values. To perform the classification, the risk associated with events occurring in the next 2 weeks was accounted as the ground truth outcome. We calculated the risk by counting the number of events during the defined time (2 weeks) and geographical (province) scales and divided it by the provincial density of poultry.

**Table 2:** Split thresholds.

Fields	VL-L	L-M	M-H	H-VH
Twitter	0	2	20	50
Spatiotemporal	− 50	0	50	100
Previous and future events	–	1	5	–

### User interface

**Figure 4:**
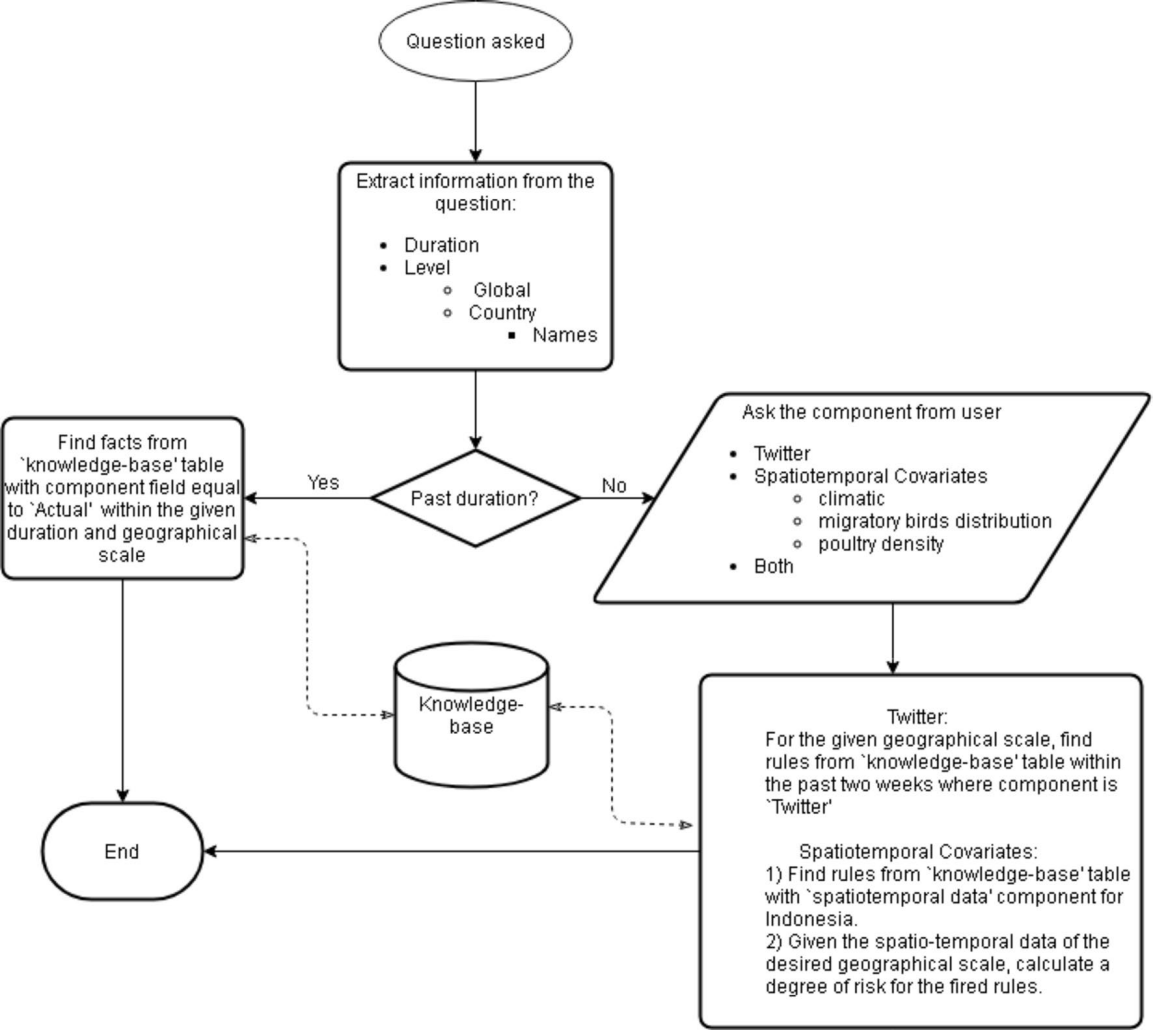
Question-answering scenarios.

The user interface part of the system can communicate between the knowledge management and user and let users specify their input and receive information. In this section, we explain how different scenarios could be followed by the proposed system in order to respond to various questions asked by end users. Considering the analytics taxonomy of descriptive, predictive and prescriptive analyses^46^, the first type of questions can be placed in the descriptive analysis group as these questions tell us what happened in the past. The second and third type of questions can be in the predictive analysis category as they forecast the future risk of disease. The process of finding answers for sample questions is given in Fig. 4.

(I) What countries have reported avian influenza in the past month? Retrospective questions could be answered using historical records of disease. We stored the facts about previous events in the ‘knowledge-base’ table and assigned the ‘Actual’ to the component field. These facts are the result of direct queries on the occurrence data and do not require any additional analyses. As depicted in Fig. 4, if the desired duration of the question is in the past, records in the table with the ‘Actual’ component field are returned.
(II) What countries are at risk of new or recurring avian influenza events in the next few weeks/Is the country X at risk of avian influenza within the next few weeks? These questions can be answered by insights that were extracted from the ‘global-scale Twitter’ component of the system. Matching rules are found based on the geographical scale (e.g. country X) and the duration of 2 weeks prior to present date (i.e. the date of question).
(III) Which provinces in country X are at risk of new or recurring avian influenza events/Is the province Y in the country X at risk of avian influenza within the next few weeks? To answer these questions, both ‘country-scale Twitter’ and ‘country-scale spatiotemporal covariates’ components could be used. Matching rules from each component return a risk degree that are then combined and used to calculate the final risk.

### Validation

The performance of decision support systems needs to be evaluated by continuous assessment of system operations. Validation of these systems is an opportunity to identify strengths and weaknesses of their functionalities and improve them. Here, the main evaluation was to ensure whether the system is meeting its goal, which is the ability to detect events. Among the questions discussed earlier, the validation of descriptive questions depends on the correctness of the collected gold standard data. Also, the validity of the second type of questions that use Twitter data to detect country-level events was already assessed^40^. The assessments concluded that 75% of real-world events were identifiable from Twitter data. The validity of the third type of questions is explained in detail as follows.

The most important element of the system validation is to identify the ability of the system in predicting the risk of future disease events. As appears in Algorithm 1, line 15, predicted risk for each week was compared to the actual risk of events. A repeated random subsampling approach was used for validation of the prediction model. The data was split into test/train datasets with a proportion of 30% for ten times. Each time, the model was built with train dataset and validated by several measures (Eqs. 1–7) on test dataset. Finally, the model was evaluated by averaging the measures. The popular accuracy measure was not considered as it could be a misleading and unreliable measure for imbalanced datasets. This is because accuracy assigns higher ranks to majority classes^47^.

Taking a class $C_i$ into consideration, the positive predictive value represents the number of correctly predicted $C_i$ out of all predicted as $C_i$. On the other hand, the sensitivity is the number of correctly predicted $C_i$ out of the number of actual examples with $C_i$ class. Specificity measures the ability of the system to correctly identify classes other than $C_i$. Finally, F-score is a weighted average of positive predictive value and sensitivity and G-mean^48^ is geometric mean of sensitivity and specificity. Also, the micro-average aggregates the contributions of all classes to compute the average metric, whereas macro-average calculates the measures independently for each class and then takes the average. In fact, the micro-average weights all examples equally and therefore, favouring the performance on major classes while the macro-average weights all the classes equally without taking the number of examples in each class into account^49^. Therefore, in the present study we consider macro-average measures in order to be able to assess the effectiveness of small classes.

Calculating measures for class $C_i$, if the actual class is $C_i$ and the predicted output is $C_i$ too, we count it as true positive (TP), and if the predicted output is a class rather than $C_i$, we count it as false negative (FN). On the other hand, assuming the actual class is a class rather than $C_i$ and the predicted output is $C_i$, we call it false positive (FP), otherwise true negative (TN).

1 $$\begin{aligned}&Positive\ Predictive\ Value\ (Precision) = \frac{TP}{(TP + FP)} \end{aligned}$$

2 $$\begin{aligned}&Sensitivity\ (Recall) = \frac{TP}{(TP + FN)} \end{aligned}$$

3 $$\begin{aligned}&Specificity = \frac{TN}{(TN + FP)} \end{aligned}$$

4 $$\begin{aligned}&F_{\mathrm{1}}-score = \frac{2 * Precision * Recall}{Precision + Recall} \end{aligned}$$

5 $$\begin{aligned}{}&G-mean = \sqrt{Sensitivity * Specificity} \end{aligned}$$

6 $$\begin{aligned}&Micro-average\ (sensitivity) = \frac{TP_{\mathrm{L}} + TP_{\mathrm{M}} + TP_{\mathrm{H}}}{TP_{\mathrm{L}}+ TP_{\mathrm{M}} + TP_{\mathrm{H}} + FN_{\mathrm{L}}+ FN_{\mathrm{M}} + FN_{\mathrm{H}}} \end{aligned}$$

7 $$\begin{aligned}&Macro-average\ (measure) = \frac{measure_{\mathrm{L}} + measure_{\mathrm{M}} + measure_{\mathrm{H}}}{3} \end{aligned}$$

## Results and discussion

### System responses examples

For the first type of questions, an example of input fields and the generated output is given in Tables 3 and 4, respectively. The facts that have been stored in the knowledge base were the result of direct queries on the datasets related to the historical record of diseases. The output of the following question is generated by finding the rows matching the given input fields. As shown in Table 3, we requested global scale events that had happened in the three past months prior to the time of question. The field ‘component’ is set to ‘actual’, which shows that the ‘actual’ component of the system was involved in answering the question. In response to the question, Table 4 was generated. The output provided information on the name of countries, the year and week when outbreaks occurred. Here, the number of outbreaks that have occurred within the defined period is indicated by field ‘value’.

Question (i): What countries have reported avian influenza in the last three months?

**Table 3:** Question (i): input fields.

Assumed current date	Duration	Weeks	Scale	Component
2019/11/23	2019/8/22–2019/11/22	34–47	Global	Actual

**Table 4:** Question (i): output.

Name	Year	Week	Unit	Value
India	2019	36	Outbreak	1
India	2019	37	Outbreak	1
Vietnam	2019	37	Outbreak	1
France	2019	41	Outbreak	1
Vietnam	2019	42	Outbreak	1
South Africa	2019	44	Outbreak	2

The second type of questions, i.e. question (ii), used the global Twitter component (see Table 5). The insights stored in the knowledge base to answer these questions have been obtained from anomaly detection analysis of global Twitter data since last 2 weeks. The generated output in Table 6 summarizes countries that have shown anomalies in their associated Twitter posts along with the time when anomalies have been seen and the number of posts (‘unit’ and ‘value’ fields).

Question (ii): What countries are at risk of new or recurring avian influenza events in the next 2 weeks?

**Table 5:** Question (ii): input fields.

Current time	Duration	Weeks	Scale	Component
2018/03/06	2018/03/07–2018/03/20	10–12	Global	Twitter

**Table 6:** Question (ii): output.

Name	Year	Week	Unit	Value
India	2018	9	Post	20
Netherlands	2018	9	Post	36
Vietnam	2018	9	Post	10
Bulgaria	2018	10	Post	179
China	2018	10	Post	223
Japan	2018	10	Post	5

The third type of questions, i.e. question (iii), finds the degree of risk for the provinces in a specified country. Given input fields provided in Table 7, both Twitter and spatiotemporal components were employed in order to calculate risks at a country scale.

Question (iii): Which provinces in Indonesia are at the risk of new or recurring avian influenza events in the next 2 weeks?

**Table 7:** Question (iii): input fields.

Current time	Weeks	Scale	Component
2016/10/29	45–46	Country	Both

**Table 8:** Question (iii): output.

Province	Week	Predicted risk
West Java	43	M
West Java	44	H
East Java	43	M
East Java	44	H
Central Java	43	H
Central Java	44	H
Bangka Belitung	43	H
Bangka Belitung	44	H
Banten	43	H
Banten	44	H
North Sulawesi	43	H
North Sulawesi	44	H
South Kalimantan	43	M
South Kalimantan	44	M

**Figure 5:**
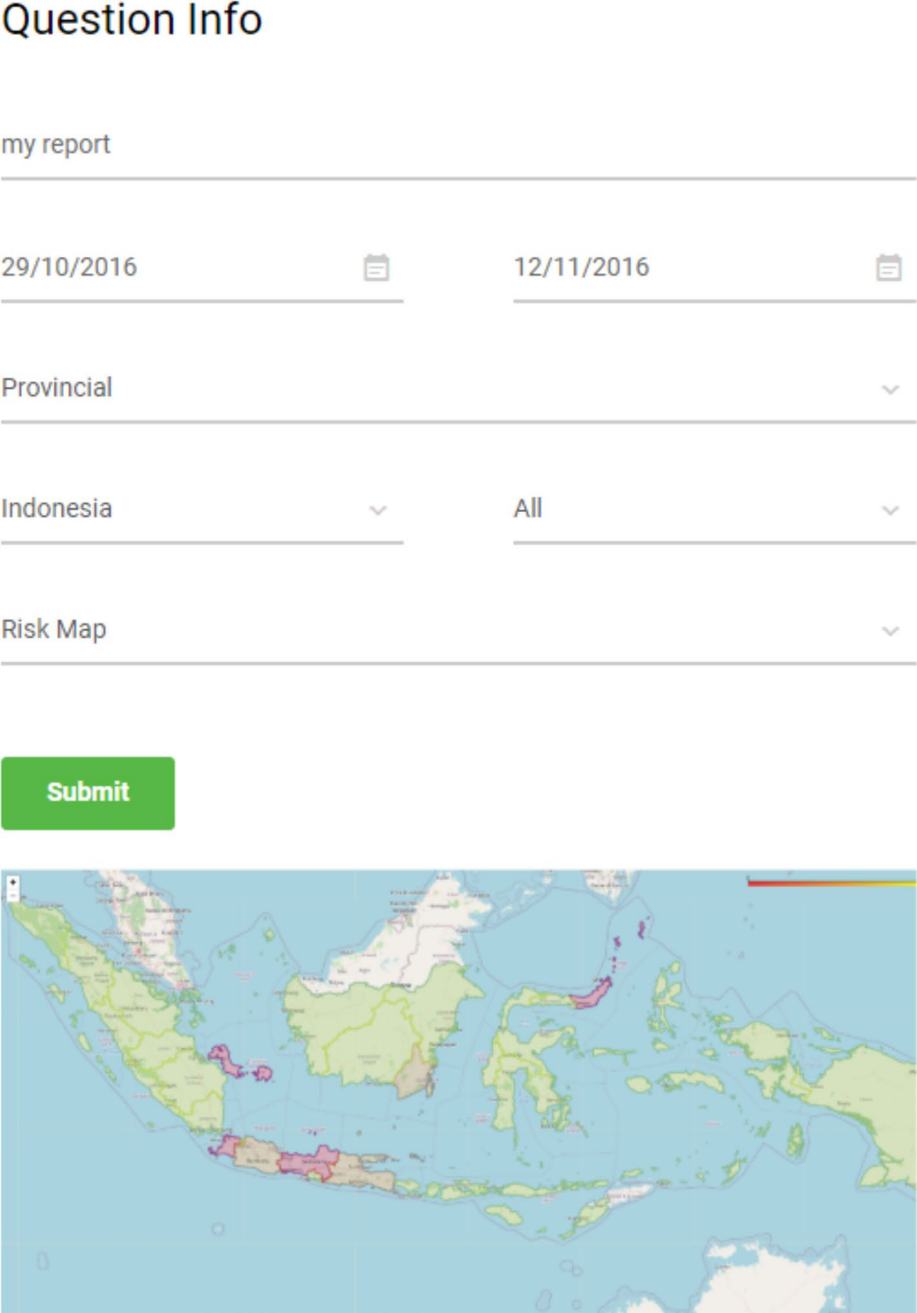
Provincial risk map (Indonesia)^26^. The figure shows the user Indonesia map with green, orange and red colors representing low, medium and high provincial risks, respectively.

The generated outcome of the third question is given in Table 8 and the user interface and outcome for week 43 are illustrated in Fig. 5. The figure shows the user Indonesia map with green, orange and red colors representing low, medium and high provincial risks, respectively.

### System assessment

To evaluate the provincial risk of disease presence, positive predictive value, sensitivity, specificity, F-score, G-mean measures (Eqs. 1–5) and their modified versions were calculated for low, medium and high categories. In the modified version of measures, the distance between predictions was taken into consideration. For example, the error associated with the prediction of low risk as the medium was calculated as half of the error associated with predicting the risk as high.

The original dataset included 1250 rows with 972 low, 212 medium and 66 high outcome labels. The data was undersampled by discarding observations related to provinces with zero or few events. This resulted in a total of 700 observations with low, medium and high labels of 449, 185 and 66, respectively.

**Figure 6:**
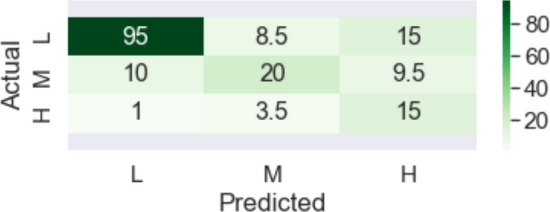
Multi-class prediction heatmap (test dataset).

A confusion matrix resulted from validation of the multi-class classification with decision tree model on testing data is reported in Fig. 6. From the confusion matrix, about 23 examples with low risk were predicted as medium or high classes, which shows false negatives for the low class. Also, 11 examples were predicted as low, while they actually belong to other classes. This represents false positives for the low class. The same approach was used to calculate false positives and false negatives for medium and high classes. The values of correctly predicted examples for each class (i.e., true positives) were placed on the diagonal line from up-left to bottom-right for the low, medium and high classes, respectively. The darker the colors get, the higher the values are.

Given the information provided by the confusion matrix, we calculated other measures and they are reported in Table 9. Notably, the meaning of positive and negative classes, which are usually used in epidemiologic studies can be switched depending on which class we select as positive. It is desirable to gain high prediction accuracy for minority class and reasonable accuracy for the majority class in risk-assessment systems^50^. In other words, although effective risk prediction methods emphasize on having less false negatives, false alarms should not be underestimated.

In general, sensitivity and specificity measures evaluate the ability of a model to predict true positives and true negatives of each class. Taking the high class as positive (minority class), a sensitivity (positive accuracy) of 78.94% and a specificity (negative accuracy) of 84.54% were achieved. This means that the model had a high ability both in predicting high-risk and non-high-risk events, but it is slightly better in predicting non-high-risk events.

Additionally, G-mean was calculated to be 81.69% for the high class, which shows the high performance of classifier simultaneously in positive and negative classes. Unlike G-mean, F-score is dependent on class distribution and is only concerned with the minority class^51,52^.

**Table 9:** Validation measures for each class.

Measure	Low (%)	Medium (%)	High (%)
Positive predictive value	81.19	45.45	30.61
Positive predictive value (modified)	89.20	62.50	37.97
Sensitivity	74.80	33.34	65.21
Sensitivity (modified)	80.16	50.00	78.94
Specificity	73.49	84.00	81.81
Specificity (modified)	80.67	91.30	84.54
F-score	77.86	38.46	41.67
F-score (modified)	84.44	55.56	51.28
G-mean	74.14	52.91	73.04
G-mean (modified)	80.42	67.56	81.69

Two types of average measures, i.e. micro-average and macro-average (Eqs. 6–7), have been commonly used as an extension of evaluation measures for multi-class classification^53^. The original and modified versions of micro-average and macro-average measures for sensitivity and specificity are reported in Table 10.

In the Table 10, the micro-average sensitivity shows a higher value than macro-average sensitivity. This is because micro-average measure favoured the majority class^54,55^ that had a higher sensitivity. Since the data is imbalanced here, the macro-average measures might be more reliable as all classes get equal weights^54^.

**Table 10:** Average measures of risk prediction.

Measure	Mic-avg (%)	Mic-avg (modified) (%)	Mac-avg (%)	Mac-avg (modified) (%)
Sensitivity	61.90	73.03	57.78	69.70
Specificity	80.95	86.51	79.77	85.50

## Conclusions and future work

Here, we described a decision support system that was designed, implemented and evaluated for monitoring and prediction of the risk of avian influenza events. The main goal of the present paper was to answer pre-defined questions asked by decision makers regarding the risk of disease at different geographical scales.

We reported evaluation measures of the prediction system overall and for each category of low, medium and high risks. Results showed relatively high macro-average measures, suggesting that the system is robust enough to be used as a decision support system in predicting avian influenza events. The evaluation measures of the high-risk class indicated that the system had lower false negative than false-positive errors while identifying high-risk events, which is preferable in risk-assessing systems.

As the framework presented here is based on questions directly asked by decision-makers, it could be a stepping stone for creating more connections between animal health officials and modellers. Unlike black-box decision support systems, the system presented here used a collection of rules as a high-level description of data, which is similar to the way humans would describe the data.

The proposed framework here employed cross-disciplinary concepts toward monitoring and prediction of avian influenza at country and global scales. The architecture allowed for a comprehensive, timely and systematic data collection, integration and analysis. Since the insights from various data analysis are continuously stored and analyzed, the time required for risk assessment is reduced, which can consequently lead to rapid decision making. To deal with uncertainty, in addition to utilizing heterogeneous data sources, the final decision tree model in the present paper calculates the probability of predictions. Moreover, a fuzzy logic rule-based framework can be designed in the future to consider the uncertainty of information using membership functions.

Importantly, the proposed framework may not predict outbreaks caused by low pathogenic viruses as accurately as the ones originated by the high pathogenic viruses. This is due to the fact that the ground truth data (OIE and EMPRES-i) only considers highly pathogenic avian influenza and low pathogenicity H5 and H7 avian influenza viruses that are notifiable.

Considering the analytic spectrum, we implemented descriptive and predictive analytics. However, the system can be enhanced by adding prescriptive analytics in the future. As an example, this will allow the system to evaluate several control policies in a high-risk area and suggest the most optimized ones to end users. Also, the applicability of the proposed framework might be enhanced by extending the database to include new data sources. Additional work might include ongoing collaboration and interactions between modellers and policy makers to constantly monitor the performance of warning systems.

## Data Availability

The datasets generated and analysed during the current study are available from the corresponding author on reasonable request.
